# *MUTYH*-associated polyposis (MAP), the syndrome implicating base excision repair in inherited predisposition to colorectal tumors

**DOI:** 10.3389/fonc.2012.00083

**Published:** 2012-08-02

**Authors:** Tiziana Venesio, Antonella Balsamo, Vito G. D'Agostino, Guglielmina N. Ranzani

**Affiliations:** ^1^Unit of Pathology, Institute for Cancer Research and TreatmentCandiolo, Torino, Italy; ^2^Department of Biology and Biotechnology, University of PaviaPavia, Italy

**Keywords:** base excision repair (BER), colorectal cancer (CRC), familial adenomatous polyposis (FAP), hereditary non-polyposis colorectal cancer (HNPCC), mismatch repair (MMR), *MUTYH*-associated polyposis (MAP)

## Abstract

In 2002, Al-Tassan and co-workers described for the first time a recessive form of inherited polyposis associated with germline mutations of *MUTYH*, a gene encoding a base excision repair (BER) protein that counteracts the DNA damage induced by the oxidative stress. *MUTYH*-associated polyposis (MAP) is now a well-defined cancer susceptibility syndrome, showing peculiar molecular features that characterize disease progression. However, some aspects of MAP, including diagnostic criteria, genotype-phenotype correlations, pathogenicity of variants, as well as relationships between BER and other DNA repair pathways, are still poorly understood. A deeper knowledge of the *MUTYH* expression pattern is likely to refine our understanding of the protein role and, finally, to improve guidances for identifying and handling MAP patients.

## Introduction

About one-third of colorectal cancers (CRC) are ascribable to a genetic predisposition. In approximately 5% of cases, CRC occurs in the context of Mendelian syndromes and is associated with highly penetrant gene mutations; in 20–25% of cases it can be considered as “familial” and likely due to low/medium-penetrant genetic variants (reviewed by de la Chapelle, 2004). More generally, all non-Mendelian CRCs can be regarded as complex diseases, where multiple genetic variants and environmental factors can modulate the disease risk. Genome-wide association studies (GWAS) have recently identified over a dozen risk loci, statistically associated with CRC (Tomlinson, 2012). The present challenge is to assess their functional significance and to clarify their interaction with probable or well-established environmental risk factors (Dunlop et al., 2012).

Among CRC predisposing syndromes, Hereditary Non-Polyposis Colorectal Cancer (HNPCC) or Lynch syndrome (OMIM#114500; NCBI database of human genes and genetic phenotype: www.ncbi.nlm.nih.gov/omim) is the most common disease; this autosomal dominant disorder is characterized by early onset CRC and extracolonic manifestations. HNPCC is associated with inherited defects in DNA Mismatch Repair (MMR) genes, primarily *MSH2* and *MLH1* (reviewed by Lynch et al., 2009). A less common syndrome is Familial Adenomatous Polyposis (FAP; OMIM#175100); this autosomal dominant condition is characterized by the development of hundred to thousands of colorectal adenomas predisposing to CCR. Congenital retinal pigment epithelial hypertrophy (CHRPE), gastric and duodenal adenomas, desmoid tumors, and thyroid cancers, are the possible extracolonic manifestations. FAP is associated with germline mutations in *APC* that encodes a tumor suppressor protein acting as a regulator of WNT signal transduction pathway. A milder form of FAP, termed attenuated FAP (AFAP), is characterized by the development of less than 100 colorectal adenomas, a more limited expression of extracolonic features, and a delay in onset of CRC; germline mutations associated with AFAP have mainly been detected in the 5′ and 3′ end of *APC*, and in the alternatively spliced exon 9 of the gene (reviewed by Jasperson et al., 2010).

In 2002, Al-Tassan and coworkers described for the first time a recessive form of polyposis associated with biallelic mutations of *MUTYH* (Al-Tassan et al., 2002; Jones et al., 2002; Sieber et al., 2003); this gene encodes a base excision repair (BER) protein that counteracts DNA damage induced by oxidative stress. This disease is currently known as MAP for *MUTYH*-associated polyposis.

## The MAP patients: phenotype, CRC risk, and surveillance

Presently, due to the variability of clinical features in *MUTYH* mutation carriers (Morak et al., 2010), the diagnostic criteria for MAP are not fully established and patients presenting FAP-like phenotypes are frequently difficult to classify. Usually, patients directed to *MUTYH* genetic testing have disease family history compatible with an autosomal recessive mode of inheritance, colorectal polyposis, and no identifiable mutations in *APC* (Sampson et al., 2003; Russell et al., 2006; reviewed by Aretz, 2010). In biallelic *MUTYH* mutation carriers the colonic phenotype resembles that of AFAP, with onset in the fourth-fifth decade and a limited number of adenomas (30-100) that increase susceptibility to CRC (Venesio et al., 2004; Filipe et al., 2009); however, unlike AFAP, hyperplastic and sessile serrated polyps can be found in MAP (Boparai et al., 2008; Zorcolo et al., 2011). Importantly, approximately 60% of MAP patients with polyposis have CRC at first presentation (mean age at diagnosis 48 years) (Nielsen et al., 2009). Moreover, a number of MAP patients with CRC and no polyps have been reported (reviewed by Nielsen et al., 2011). In a population-based screening, the penetrance of CRC has been estimated 20% and 43% at 50 and at 60 years of age, respectively (Lubbe et al., 2009). Since oxidative stress occurs in various cell types, *MUTYH* inactivation can be expected to predispose not only to intestinal, but also to extraintestinal lesions. Key extracolonic manifestations include predisposition to duodenal adenomas and cancer (Nielsen et al., 2006); in addition, constitutive gene mutations have been detected in patients with endometrial carcinoma (Barnetson et al., 2007; Tricarico et al., 2009). A recent multicenter study showed that the incidence of extraintestinal malignancies among MAP cases is almost twice that of the general population, with a significant increase in the incidence of ovarian, bladder, and skin cancers, and a trend of increased risk of breast tumors; interestingly, this cancer spectrum overlaps with HNPCC syndrome (Vogt et al., 2009). Thyroid carcinomas have been documented; however, in contrast to FAP, MAP does not appear to be associated with this cancer type (Ponti et al., 2005; Vogt et al., 2009; Pervaiz et al., 2010).

While the strong impact of biallelic *MUTYH* mutations on CRC risk has been demonstrated, the cancer risk associated with germline monoallelic mutations is still controversial. Initially, heterozygous germline mutations had been reported to increase the risk of CRC later in life without apparent excess risk of polyposis (Farrington et al., 2005), and both biallelic and monoallelic mutation carriers had been reported more likely to have first/second-degree relatives with CRC compared with non-carriers (Croitoru et al., 2004). However, subsequent meta-analyses have produced conflicting results, indicating both a non-significant and a significant increased risk of CRC for monoallelic mutation carriers (Peterlongo et al., 2006; Tenesa et al., 2006; Webb et al., 2006). More recently, the analysis of MAP family members confirmed previous data that monoallelic carriers have a two-fold increase of risk of CRC (Jones et al., 2009; Win et al., 2011); analogous results have been obtained through a systematic evaluation of clinicopathologic/epidemiologic and genetic data in a series of CRC cases and controls from a multisite CRC registry (Cleary et al., 2009).

A large population-based series of patients and controls has been screened for the presence of the two most common *MUTYH* mutations in MAP (i.e., Y179C and G396D): following genotype-phenotype correlation, the evaluation of genotype-specific CRC risk indicated that monoallelic mutation status is not clinically relevant (Lubbe et al., 2009). Accordingly, a marginal monoallelic effect has been reported in a recent study aimed at refining the estimates of CRC risk associated with mono- and bi-allelic *MUTYH* mutations (Theodoratou et al., 2010).

During a workshop held in Mallorca in 2006 and 2007, European experts on hereditary gastrointestinal cancer established recommendations for the clinical management of polyposis. According to these guidelines, patients with more than 10 adenomas should be referred for genetic counselling, and *MUTYH* mutation analysis should be performed. The screening should start between 18 and 20 years, the same age as recommended in AFAP. Since patients frequently develop only a few adenomas and CRC is often localized in the proximal colon, colonoscopy at 2-yearly intervals instead of sigmoidoscopy should be performed. Due to the relatively high risk for duodenal cancer, upper gastrointestinal endoscopy should start between 25 and 30 years of age. If surgery is required, IRA is sufficient in most cases to eliminate cancer risk (Vasen et al., 2008).

Recently, Nieuwenhuis and collaborators (2012) underlined the high risk in MAP patients to develop CRC even under surveillance, suggesting colonoscopy at short intervals (1–2-year intervals) starting from the age of 18–20 years, and recommending (sub)total colectomy when surgery for CRC is justified.

In the absence of clear-cut data on monoallelic *MUTYH* mutation carriers, CRC surveillance should follow guidelines proposed for subjects with a family history of CRC, without intensive screening, i.e., colonoscopy starting at age 40 and repeated every five years (Levin et al., 2008).

The lifetime risk for extracolonic cancers seems considerable; however, it is uncertain if more aggressive cancer surveillance for these lesions than is recommended for the general population would be valuable (Nielsen et al., 2009; Terdiman, 2009).

## Molecular mechanisms underlying MAP

Al-Tassan and colleagues (2002) reported for the first time that predisposition to multiple colorectal adenomas and carcinomas can be inherited in an autosomal recessive manner. By analyzing tumor DNAs from three affected individuals of the same family, they found that *APC* tumor suppressor gene was somatically inactivated due to the frequent occurrence of G:C to T:A transversions. Such a pattern was suggestive of a defect in the repair system that prevents mutations caused by spontaneous oxidation of guanine to 8-oxoguanine, the most prevalent product of the oxidative stress (reviewed by David et al., 2007). Accordingly, the human genes encoding for the enzymes of the BER pathway were entirely sequenced and germline biallelic mutations in *MUTYH* were detected in the affected members of the family. Subsequent studies have unequivocally established the association between *MUTYH* germline mutations and predisposition to adenomas and CRCs, confirming the preponderance of G:C to T:A transversions as a molecular feature of MAP-associated tumors (reviewed by Lipton and Tomlinson, 2004, and by Sampson et al., 2005).

In addition to transversions along *APC* resulting in stop codon formation and gene inactivation, somatic GGT to TGT transversions, giving rise to the G12C *KRAS* activating-mutation, were frequently detected in MAP CRCs (Lipton et al., 2003; Jones et al., 2004; Nielsen et al., 2009). Boparai and collaborators (2008) reported that not only hyperplastic polyps (HPs) and sessile serrated adenomas (SSAs) can be a typical expression of MAP, but that they also have a characteristic molecular background. In particular, *KRAS* gene mutations were shown to be present in 70% of HP/SSA in MAP patients, compared with 17% of HP/SSA in sporadic cases. Of relevance, G:C to T:A transversions accounted for 94% of the mutations in MAP-HP/SSA, with respect to 29% in sporadic HPs/SSAs. Moreover, comparing adenomas and HPs/SSAs in MAP patients, *APC* mutations were only detectable in adenomas, indicating two possible tumor pathways, one leading to adenomas *via APC* mutations, and the other leading to HPs/SSAs *via KRAS* activation.

On the whole, MAP cancers appear to follow a distinct progression pathway compared to pathways occurring in CRCs, *i.e*., with either chromosomal instability (CIN) or with high-frequency microsatellite instability (MSI-H). However, some features overlap with CIN phenotype, including frequent *APC*/*KRAS* mutations, while others with MSI-H phenotype, including unfrequent LOH at *APC* locus and near-diploid karyotype (Lipton et al., 2003; Johnson et al., 2005). Tumors from both monoallelic and biallelic *MUTYH* mutations carriers often show low-frequency microsatellite instability (MSI-L), suggesting functional interactions between BER and MMR systems (Cleary et al., 2009). Interestingly, HLA class I expression loss has been reported in both MAP and MMR-deficient tumors (De Miranda et al., 2009), suggesting that the mutagenic background of these tumor types triggers the generation of aberrant peptides likely acting as tumor neo-antigens. The elicited immune reaction might then selectively favor the outgrowth of cancer cell clones that have lost HLA class I expression, avoiding cancer cell recognition and elimination by the immune system (De Miranda et al., 2009).

In sporadic carcinogenesis, the issue of a possible role of *MUTYH* has been addressed by Halford and collaborators (2003) by screening a large sample of sporadic CRCs for somatic alterations of *MUTYH*, as well as of *MTH1* and *OGG1* BER genes: somatic inactivation of these genes does not appear a frequent mechanism directly involved in colorectal tumorigenesis. On the contrary, a reduced expression of *MUTYH* has been reported in human gastric cancer, where reduction in protein amount proved to be associated with a poor prognosis (Shinmura et al., 2011).

## The *MUTYH* gene

The *MUTYH* gene (previously termed *MYH* or *hMYH*), the human ortholog of the *Escherichia coli mutY*, was first cloned by Slupska and coworkers in 1996; it is about 11,200 bp in length, is localized on the short arm of chromosome 1 (1p32.1-p34.3), and contains 16 exons (NCBI Genomic refseq ID seq ID: NG_008189.1).

Many *MUTYH* genetic variants have been reported, having or likely not having a phenotypic effect; in this regard, the Leiden Open Variation Database represents an extremely valuable tool to evaluate gene mutations that have been identified both in healthy and affected subjects (http://chromium.liacs.nl/LOVD2/colon_cancer/home.php?action=switch_db).

**Figure 1:**
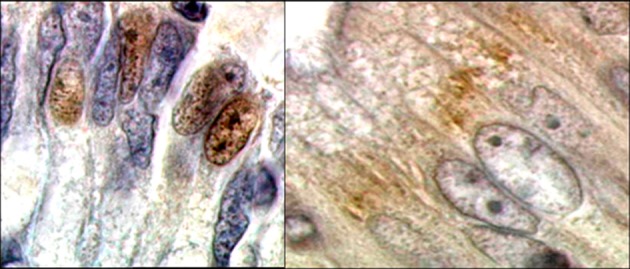
**Diffuse localization of wild-type MUTYH protein in colonic epithelial cells, as evidenced with specific antibodies (Courtesy of Dr. M. Risio)**.

*MUTYH* encodes a DNA glycosylase that is expressed both in the nucleus and in the mitochondria. The coding sequence generates three classes of mRNAs (namely α, β, and γ) that correspond to a total of 10 possible mature transcripts; these transcripts are produced by three independent transcription initiation sites and by the occurrence of different exon 3 alternative splicing events, and correspond to seven protein isoforms (Ohtsubo et al., 2000; reviewed by Parker and Eshleman, 2003). In accordance with the Human Genome Variation Society nomenclature, the longest transcript (transcript <5: 1945 bp) is used as coding reference sequence (NCBI transcript refseq ID: NM_001128425.1). Transcripts starting from the first AUG retain the sequence that corresponds to the N-terminal aminoacids likely acting as mitochondrial targeting signal (MTS) (Ohtsubo et al., 2000). The relative amounts of each of the ten possible mRNAs in different tissues as well as their subcellular localization are only partially characterized. Among different human tissues, the largest total amount of mRNA has been observed in thymus, adult brain, testis, and kidney, whereas heart, salivary gland, liver, and pancreas show lower *MUTYH* expression levels compared to other tissues (Ohtsubo et al., 2000). Very recently, it has been reported that not only *MUTYH* expression is different in various organs and positively correlated with proliferative activity, but also the first exons of the gene are used in a tissue-specific manner. In addition, transcripts encoding mitochondrial proteins have been shown to predominate in muscle tissues, while the highest amount of transcripts encoding nuclear proteins have been detected in testes and colon (Plotz et al., 2012). As example of protein expression, Figure 1 shows the diffuse localization of MUTYH in normal cells of colon epithelium.

## The BER system and the MUTYH protein

A number of deleterious processes and pathological conditions may result from DNA oxidation, including initiation and progression of cancer. DNA oxidation arises from its interaction with exogenous molecules or from the action of reactive oxygen species (ROS); these can be generated by the metabolism of exogenous compounds as well as by cellular processes including respiration and inflammation.

The BER system is a cellular defense against the damaging effects of ROS. Generally, BER is initiated by damage-specific DNA glycosylases that recognize oxidized bases and excise the damaged nucleotides, allowing the restoration of the parental DNA sequence by further processing of endonucleases, polymerases, and ligases (reviewed by Fortini and Dogliotti, 2007; van Loon et al., 2010). A very well known oxidation product is the highly mutagenic 7,8-dihydro-8-oxoguanine (8-oxoguanine or 8-oxoG) which is able to form both 8-oxoG:C and 8-oxoG:A stable base pairs. Failure to remove the incorporated 8-oxoG before the next round of DNA replication results in G:C to T:A transversion mutations (reviewed by David et al., 2007).

The occurrence of 8-oxoG in the DNA arises from two pathways: incorporation into DNA of the oxidized precursor 8-oxo-dGTP during DNA synthesis and direct oxidation of guanine in DNA. The 8-oxo-dGTP can be removed from the nucleotide pool by the oxidized purine nucleoside triphosphatase MTH1, which prevents the selection of the oxidized guanine by polymerases during DNA replication. In addition to this sanitizing mechanism, two other BER enzymes act as second line of defense, namely OGG1 and MUTYH. OGG1 has a high specificity for the 8-oxoG:C and removes the oxidized guanine, allowing restoration of a G:C base pair, while MUTYH is a DNA glycosylase able to recognize and remove either adenine from a mismatch with 8-oxoG, or 2-OH-adenine from a mismatch with guanine (reviewed by Nakabeppu et al., 2004; Ushijima et al., 2005).

MUTYH is the functional counterpart of *E. coli* MutY and is a highly evolutionarily conserved protein; it belongs to a large superfamily of structurally related DNA glycosylases with a signature helix-hairpin-helix motif followed by a Gly/Pro-rich (denoted as HhH-GPD domain) region. This domain contains the active site pocket responsible for the 8-oxoG:A mispair recognition and for the glycolytic excision of the substrate adenine (Lee and Verdine, 2009). MUTYH shows an N-terminal domain containing, apart from the mitochondrial localization signal (MLS) and a putative nuclear localization signal (NLS), the replication protein A (RPA) interacting motif; the C-terminal domain contains a NLS and the proliferating cell nuclear antigen (PCNA) binding region. The interaction with PCNA is crucial in replication-coupled repair in order to increase and direct the glycosylase activity of MUTYH to the newly synthesized nascent DNA strand, thus preventing replication errors caused by a 8-oxoG template (Hayashi et al., 2002; reviewed by Parker and Eshleman, 2003; van Loon et al., 2010). *In vitro* experiments have shown that MUTYH physically interacts with the MSH2/MSH6 heterodimeric complex *via* the MSH6 subunit, demonstrating that this MMR protein complex stimulates the glycosylase activity of MUTYH by enhancing the protein affinity for the 8-oxoG:A mismatches (Gu et al., 2002; Bai et al., 2005). Besides multiple interactions with replication and repair proteins, MUTYH activity can also be modulated by post-translational modifications that regulate protein localization and level (Hirano et al., 2003).

The 8-oxoG accumulation in nuclear and in mitochondrial DNA triggers two distinct cell death pathways that, although independent of each other, are both associated with BER and initiated by MUTYH activity (Ichikawa et al., 2008; Oka et al., 2008). Oka and coworkers (2008) demonstrated that the knockdown of *MUTYH* results in escaping from both types of pathways and proposed a tumor suppressor role of MUTYH due to its capability to induce death of pre-cancerous cells that have accumulated high levels of 8-oxoG in nuclear or in mitochondrial DNA.

ROS can be produced by the cellular components of the inflammation; oxidative stress and consequent DNA damage play a key role in the pathogenesis of ulcerative colitis (UC), a form of inflammatory bowel disease characterized by an increased risk of CRC. In particular, the UC-associated tumors display both an accumulation of 8-oxoG and an altered expression of MUTYH protein in cancer cells (Gushima et al., 2009). Accordingly, in a mouse model of UC, *Mutyh* has recently been shown to play a major role in maintaining intestinal integrity by influencing the inflammatory response (Casorelli et al., 2010).

## Germline mutations of *MUTYH* in MAP patients

Approximately 30% of *APC* mutation-negative polyposis cases can be attributed to *MUTYH* biallelic mutations; on the whole, these account for less than 1% of all CRC cases (reviewed by Cheadle and Sampson, 2007; Cleary et al., 2009). To date, nearly 300 variants have been identified at the *MUTYH* locus, including about 80 pathogenic mutations distributed throughout the gene and located at positions corresponding to different functional domains of the protein (see: Leiden Open Variation Database). Although various types of alterations have been reported in MAP patients, including nonsense, small insertion/deletion, and splicing variants, missense mutations represent the great majority of the detected changes. A number of variants appear recurrent in different populations, with Y179C (previously annotated as Y165C) and G396D (previously annotated as G382D) missense mutations accounting together for about 70% of germline alterations found in European patients (reviewed by Cheadle and Sampson, 2007); however, in Asian populations, Y179C and G396D must be rare, since neither mutation has been found in MAP patients. On the other hand, other mutations have proven to be recurrent in patients from particular populations (reviewed by Poulsen and Bisgaard, 2008). Taken together, these findings indicate that sequencing of the entire *MUTYH* open reading frame has to be performed for the genetic testing, especially in populations of mixed ethnicity.

Recently, a large gene deletion spanning exons 4–16 has been found in two unrelated patients showing an attenuated phenotype; due to this observation, appropriate methods to detect gene rearrangements should be considered, at least for patients carrying either a single heterozygous mutation or a (apparently) homozygous disease-causing mutation (Rouleau et al., 2011; Torrezan et al., 2011). Besides rare mutations, a polymorphic allele (SNP rs3219468: G>C) associated with a significant reduction of a *MUTYH* transcription product has recently been implicated in CRC risk (Plotz et al., 2012).

The identification of germline mutations in patients with inherited CRC syndromes, including MAP, is extremely important to allow mutation carriers to be included in cancer surveillance programs which have been proven to save lives. Many of the identified disease-gene mutations result in loss-of-function of the encoded protein, indicating a clear pathogenic significance. However, significance remains uncertain for a large proportion of the identified variants, some of which may contribute to increase the risk of cancer. For MAP, the major problem is represented by missense mutations, the pathogenetic role of which cannot easily be assessed; this leaves open questions about their diagnostic interpretation and about counseling management of the patients in whom they are detected.

**Figure 2:**
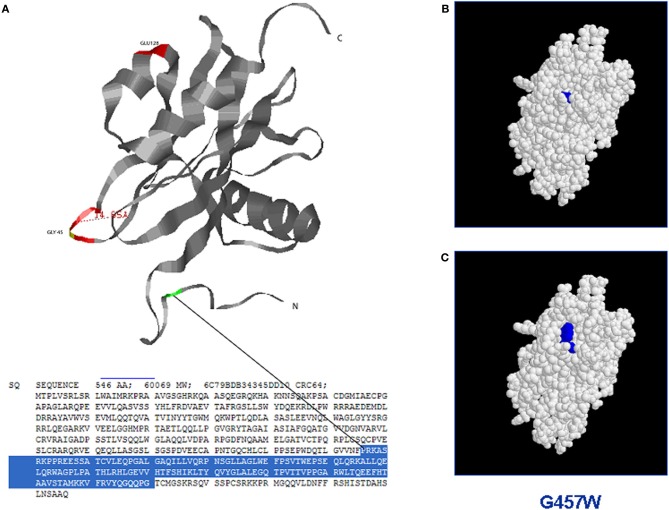
**Structure-based analysis of mutant MUTYH protein by homology modelling (*Site Directed Mutator*): the figure shows the possible switch of the MUTYH protein folding when a single aminoacid residue is mutated in the NUDIX domain. (A)** Three dimensional structure of the NUDIX domain of MUTYH protein (*PDB database*); **(B)** Protein folding of the wild-type NUDIX domain. **(C)** Protein switch of the NUDIX domain containing the substitution G457W.

Since MUTYH mitigates the mutagenic potential of 8-oxoG by preventing accumulation of transversion mutations, *mutY*-deficient *E. coli* cells have been used to study if the human heterologous mutated proteins have the ability to complement the mutator phenotype in these cells (Chmiel et al., 2003; Bai et al., 2005, 2007). Analogously, to characterize the functional effect of some missense mutations, human proteins have been expressed in *Mutyh*-defective mouse fibroblasts and their biological activity has been related to the 8-oxoG level in the genome and to the response of cells following oxidative stress induction (Molatore et al., 2010). Further, lymphoblastoid cell lines derived from MAP patients have been established and immunoprecipitated proteins from whole cell extracts have been tested *in vitro* (Alhopuro et al., 2005; Parker et al., 2005). Owing to the capacity of MUTYH to recognize and excise adenine from DNA duplexes containing either 8-oxoG:A or G:2-OH-A mismatches, *in vitro* tests with purified proteins have been performed. By using different oligonucleotides containing specific mismatches as substrate, the enzymatic activities of bacterial (Al-Tassan et al., 2002; Chmiel et al., 2003; Livingston et al., 2005), mouse (Hirano et al., 2003; Pope et al., 2005; Ushijima et al., 2005; Yanaru-Fujisawa et al., 2008), and human (Bai et al., 2005, 2007; Ali et al., 2008; Kundu et al., 2009; D'Agostino et al., 2010) purified proteins have been investigated. The glycosylase activity has been evaluated by measuring the amount of cleavage fragments derived from DNA duplexes, while the DNA-binding capacity has mainly been investigated through the formation of DNA-protein complexes in electrophoresis mobility shift assay (EMSA). Since surface plasmon resonance (SPR) technology is extremely sensitive to detect subtle and time-dependent changes in DNA-protein interaction and to investigate the enzymatic activity on different DNA substrates, this method has successfully been used to evaluate the binding of some mutated human MUTYH proteins to an 8-oxoG:A-containing DNA substrate (D'Agostino et al., 2010). Recently, computational methods based on amino acids substitutions within homologous proteins with known 3D structures have been applied to predict changes of different proteins, thus correlating amino acids substitutions with functional effects possibly resulting in phenotype changes (Worth et al., 2007). As for other proteins, the understanding of the mechanisms by which mutations affect MUTYH protein structure can provide new insights into its activity and, ultimately, on the pathogenicity of gene mutations. Figure 2 shows an example of the structure-based analysis of MUTYH mutated proteins.

On the whole, the majority of MUTYH tested variants have been found to be totally or partially devoid of DNA-glycosylase, with variable adenine-removal residual capability. As an example, functional assays demonstrated that G396D capability is far more similar to the wild-type protein compared to Y179C missense mutation. Accordingly, clinical data indicate a milder phenotypic effect of G396D compared to Y179C. Indeed, Nielsen and collaborators (2009) reported that MAP patients with homozygous G396D mutations or with compound heterozygous G396D/Y179C mutations show a milder phenotype when compared to homozygous carriers of Y179C mutations.

The expression pattern of MUTYH mutated proteins has been evaluated on CRC tissue sections to test whether immunohistochemistry can be used in clinical practice to identify carriers of germline mutations with functional effects. However, the specificity of the expression pattern has proven to be uncertain and immunostaining does not seem an appropriate tool to discriminate CRC tissues from patients with or without biallelic germline mutations (Di Gregorio et al., 2006; van der Post et al., 2009).

## Concluding remarks

During the last 10 years MAP has clearly become a distinct cancer-susceptibility syndrome with respect to both FAP and AFAP. However, in order to refine clinical guidances, some aspects still poorly understood that concern the function of MUTYH protein deserve to be elucidated. Unsolved problems deal with genotype–phenotype correlations, pathogenicity of unclassified variants, cross-talk between BER and other DNA repair pathways including MMR, overlapping between MAP and HNPCC syndromes, and last, but not least, tumor suppressor role of MUTYH in pre-cancerous cells subjected to oxidative stress. A better understanding of the regulation of MUTYH isoforms, of their relative expression in different tissues, and of their subcellular fate, will likely allow us to clarify some of the above issues. By taking into account the complexity of the expression pattern, the effect on splicing efficiency and accuracy should be evaluated for a number of *MUTYH* variants that have been identified in patients without clear disease-causing mutations. Indeed, a fraction of gene mutations, including synonymous nucleotide changes, have been shown to have roles in various diseases or to modify disease severity through splicing perturbation rather than other molecular mechanisms (reviewed by Cartegni et al., 2002; Wang and Cooper, 2007).

### Conflict of interest statement

The authors declare that the research was conducted in the absence of any commercial or financial relationships that could be construed as a potential conflict of interest.

## Abbreviations

MAP: *MUTYH*-associated polyposis
CRC: colorectal cancer
HNPCC: hereditary non-polyposis colorectal cancer
FAP: familial adenomatous polyposis
BER: base excision repair
MMR: mismatch repair
OMIM: Online Mendeleian Inheritance in Man.
